# Improvement of photosynthesis in rice (*Oryza sativa* L.) by inserting the C4 pathway

**DOI:** 10.1186/1939-8433-6-28

**Published:** 2013-10-28

**Authors:** Shanta Karki, Govinda Rizal, William Paul Quick

**Affiliations:** C4 Rice Center, International Rice Research Institute, Los Banos, Laguna Philippines; Department of Animal and Plant Sciences, University of Sheffield, Sheffield, UK

**Keywords:** Bundle sheath, Chloroplast, Mesophyll, Photorespiration, Photosynthesis

## Abstract

To boost food production for a rapidly growing global population, crop yields must significantly increase. One of the avenues being recently explored is the improvement of photosynthetic capacity by installing the C4 photosynthetic pathway into C3 crops like rice to drastically increase their yield. Crops with an enhanced photosynthetic mechanism would better utilize the solar radiation that can be translated into yield. This subsequently will help in producing more grain yield, reduce water loss and increase nitrogen use efficiency especially in hot and dry environments. This review provides a summary of the factors that need to be modified in rice so that the C4 pathway can be introduced successfully. It also discusses the differences between the C3 and C4 photosynthetic pathways in terms of anatomy, biochemistry and genetics.

**Electronic supplementary material:**

The online version of this article (doi:10.1186/1939-8433-6-28) contains supplementary material, which is available to authorized users.

## Review

### Introduction

To provide adequate food and nutrition to the global population that is expected to reach 9 billion by 2050 (http://www.unpopulation.org), rice yields need to increase by at least 60% (FAO 2009). Rice is the staple food of over half of the world’s population and this rice consuming population is increasing at the rate of 1.098% per annum (http://esa.un.org/wpp/Excel-Data/population.htm). Escalating population means more demand for food, water and land at a time when the natural resource base for agriculture is being degraded because large areas of farmland are being diverted from food production to industrialization and bio-fuel production. An unpredictable climate change is threatening to further reduce agriculturally viable land due to more instances of drought and flood (http://www.fao.org/docrep/017/aq191e/aq191e.pdf). As a growing population and global climate change place increasing pressure on the world’s food supply, it is essential that we continue to improve crop performance in terms of grain productivity to keep pace with population growth. The increase in crop productivity conferred by the plant types created during green revolution period supported the population boom following the two world wars. Since then, despite the use of improved varieties and advanced technologies, the yield potential of present day rice cultivars has improved just a little indicating that these varieties have hit a yield ceiling (Akita 1994). Recently an attempt is underway to increase the rice yield potential by engineering an efficient C4 type photosynthesis into rice (Kajala et al. 2011). For this, a set of genes which regulate leaf anatomy and biochemical processes have to be inserted into rice and expressed in an appropriate manner which is currently not possible solely by conventional plant breeding techniques. Therefore, genetic engineering to improve the photosynthetic pathway of rice would provide sufficient opportunity to enhance the actual grain productivity as well as the yield potential. Genetic engineering provides an efficient and precise breeding tool in which only the genes of interest can be introduced even from distantly related species.

In C3 plants like rice, CO_2_ is assimilated into a 3-carbon compound by the photosynthetic enzyme ribulose-1, 5-bisphosphate carboxylase oxygenase (Rubisco). As the name indicates, Rubisco also catalyzes oxidation of ribulose-1, 5-bisphosphate (RuBP) in a wasteful process known as photorespiration which can incur a loss of upto 25% of previously fixed carbon (Sage 2004). At temperature above 30°C which is typical of tropical rice growing areas of the world, rate of oxygenation increases substantially and this considerably reduces the photosynthetic efficiency of C3 plants by upto 40% (Ehleringer and Monson 1993). Thus, photosynthesis of rice in the tropics and warm temperate regions becomes inefficient. The C4 plants which have CO_2_ concentrating mechanism within their leaves have very much reduced levels of photorespiration and thus have evolved to thrive in hot, arid environments and offer valuable insights for crop improvement strategies. Rice with a C4 photosynthesis mechanism would have increased photosynthetic efficiency while using scarce resources such as land, water, and fertilizer specifically nitrogen more effectively (Hibberd et al. 2008). Because it will perform well under high temperature as well as require less water and nitrogen, C4 rice would confer benefits on different types of rice ecosystems including the marginal lands.

C4 type photosynthesis is one of the three types of biochemical mechanisms adopted by plants to fix atmospheric CO_2_, others being C3 and Crassulacean acid metabolism (CAM) pathways. C4 photosynthesis has evolved more than 66 times independently (Sage et al. 2012) at least in 19 families during angiosperm evolution from C3 ancestors (Muhaidat et al. 2007) and it entails alternations of cellular structures, biochemistry and hence the development of leaves. This highly specialized form of photosynthesis essentially has developed a CO_2_ concentrating mechanism around the Rubisco enzyme thus eliminating the oxygenase function of Rubisco thereby reducing the wastage of energy due to photorespiration (Douce and Heldt 2000). Rubisco from C4 species is more efficient than from C3 species in terms of carboxylation (Kubien et al. 2008). The other associated benefits of the C4 system include higher water use efficiency because steeper concentration gradient for CO_2_ diffusion can be maintained through partly closed stomata, higher radiation use efficiency as C4 photosynthesis efficiency does not get saturated at high light intensity (Rizal et al. 2012) and higher nitrogen use efficiencies because it will require less Rubisco and hence less nitrogen.

C4 plants are potentially more productive at higher temperatures typically experienced by rice. To take advantage of this more efficient photosynthetic system at a time when the population and food prices are soaring, there are efforts towards inserting the C4 mechanism such as that found in maize into rice (Rizal et al. 2012). This novel approach to modify the photosynthesis system of rice is a challenging and long term endeavor because the C4 pathway is very complex and many factors controlling the mechanism are still unknown. Therefore, it requires ingenuity and expertise of scientists involved in diverse disciplines such as genetic engineering, biochemistry, bioinformatics, molecular biology, photosynthesis, systems biology, physiology, plant breeding, metabolomics, etc. For the same, the C4 rice consortium was conceptualized and established which began the practical work of C4 rice engineering since 2009 (http://photosynthome.irri.org/C4rice/). This review provides an update on the requirements to develop C4 rice and progress made in the field of genetic engineering. Based on the study of the evolution of C4 from C3 species and the associated changes, the following modifications are essential to establish a functional C4 photosynthetic pathway in rice.

### Increase the number and size of chloroplasts in bundle sheath cells of rice

**Figure 1:**
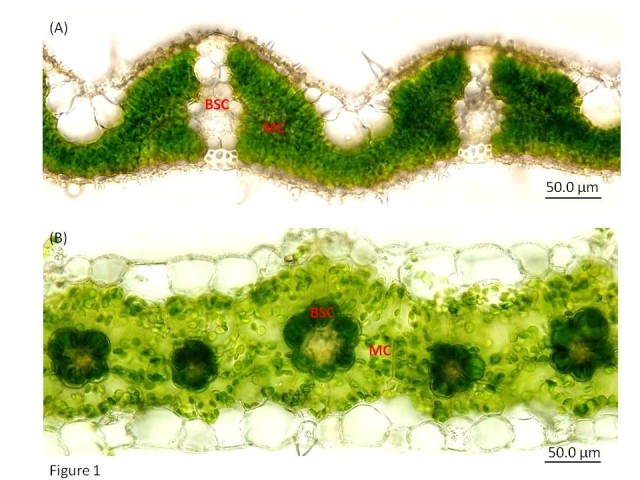
**Anatomical differences between C3 and C4 leaves. (A)** C3 (*Oryza sativa* L., rice variety IR64) and **(B)** C4 (*Setaria viridis*) leaf. Mesophyll cell (MC) of rice is filled with chloroplasts which is more than 90% of the total chloroplasts, whereas, the bundle sheath cells (BSC) have very few number of chloroplasts which account for less than 10% of the total chloroplasts in the rice leaves. In C4 leaf, chloroplasts are localized in BSC as well as in MC.

In rice more than 90% of the total chloroplasts are located in mesophyll cells (MCs) within the leaf (Yoshimura et al. 2004); whereas, in C4 plants both MCs and bundle sheath cells (BSCs) possess equal numbers of chloroplasts (Figure 1A and B). This is because in C3 plants, the entire process of photosynthesis takes place in MC, but in C4 plants the process of photosynthesis is compartmentalized into MC and BSC. The MCs perform the first CO_2_ fixation in which 4-carbon compound called oxaloacetate is formed and this is converted to C4 acids such as malate which get transported into BSCs thereby enabling efficient assimilation of CO_2_ into carbohydrates by the Calvin cycle in BSCs. Therefore, unlike in C3 plants, the BSCs of C4 plants have photosynthetic functions such as the decarboxylation of the C4 compound and the process of the Calvin cycle. To perform these processes, the BSCs in C4 plants are enlarged and have more chloroplasts, thereby making the BSCs more pronounced and photosynthetically active. The BSCs in C3 species function to balance hydraulic pressure, prevent entry of air from intercellular spaces to the xylem, provide a reservoir of water to buffer losses due to transpiration, allow entry and dispersion of higher intensity of light that hits the veins into the leaf (Nikolopoulos et al. 2002). Additional functions of BSCs of C3 plants include transport of nitrogen, sulphur, carbohydrate and role in signaling pathway which has been extensively reviewed in (Leegood 2008). In C4 species, BSCs and MCs cooperate in a two-step version of the photosynthesis. As a result, to ensure a direct contact between BSCs and MCs, C4 plants possess a special kind of leaf anatomy accompanied by proliferation of chloroplasts in BSCs. To introduce the C4 pathway into rice, more photosynthetic chloroplasts are required in the BSCs than rice has now. This could be done by over expressing the genetic elements that are necessary for the chloroplast development such as *Golden2-like* (*GLK*) genes in a cell specific manner by using C4 gene promoters such as phospho*enol* pyruvate carboxylase (PEPC) of *Zea mays* for MC specific expression and phospho*enol* pyruvate carboxykinase (PCK) promoter of *Zoysia japonica* for BSC specific expression in rice leaves (Matsuoka et al. 1994; Nomura et al. 2005).

*Golden2-like* (*GLK*) gene family members encode nuclear transcription factors that have been implicated to regulate chloroplast development in Arabidopsis, *Zea mays*, and the moss *Physcomitrella patens* (Rossini et al. 2001). In each of these species, *GLK* genes exist as a homologous pair named as *GLK1* and *GLK2* (Waters et al. 2009). In moss and Arabidopsis the *GLK* genes are redundant and functionally equivalent whereas in maize and sorghum *GLK* genes act in a cell-type-specific manner to direct the development of dimorphic chloroplasts (Waters et al. 2008; Wang et al. 2013a). In maize, *Golden2* (*G2*) and its homologue *ZmGLK1* transcripts accumulate primarily in BS and M cells, respectively, suggesting a specific role for each gene regulating the dimorphic chloroplast differentiation (Wang et al. 2013a).

### Reduce the vein spacing thereby increasing the vein density in the leaf

**Figure 2:**
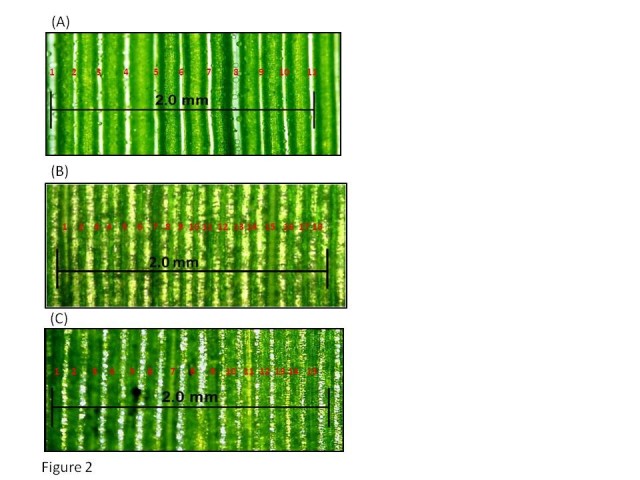
**Variation in leaf vein density between C3 and C4 plants.** Vein density of **(A)** C3 (*Oryza sativa* L., rice variety IR64), **(B)** C4 (*Setaria viridis*) and **(C)** C4 (*Sorghum bicolor*) leaf sections. Rice has low vein density compared to the C4 plants like *S. viridis* and sorghum.

In C3 species, photosynthesis takes place in the MCs. High numbers of MCs between the consecutive veins (Figure 1A) pushes the veins far from each other thus increasing the vein spacing or reducing the vein density. In rice leaves there are less than 6 veins per mm (Figure 2A), *Setaria viridis* and sorghum (both are typical C4 species) have more than 7 veins per mm (Figure 2B and C). C4 leaves on average have 2 MCs between the veins (Figure 1B). The higher vein density in the leaves of C4 plants leads to a nearly one-to-one ratio of the volumes of M and BS tissues. The internal anatomy of a C4 leaf is often composed of a repeating pattern of vein-BS-M-M-BS-vein. BSCs surrounded by MCs form a wreath-like structure; this type of leaf anatomy was termed as “Kranz anatomy” by the German botanist G. Haberlandt. C4 BSCs have dense cytoplasm and are filled with large numbers of chloroplasts (Figure 1B). For the efficient functioning of the C4 pathway, a close contact between M and BS cells is indispensable and these are tightly interconnected to each other with large numbers of plasmodesmata Dengler and (Nelson 1999). Kranz anatomy is found with little variation in nearly all monocotyledonous and dicotyledonous lineages that use the two-cell mode of C4 photosynthetic pathway. Studies on leaf anatomy and morphology have revealed several genes responsible for growth, development or deformities of cells in leaves. A gene *ACAULIS1* was responsible for elongation of leaf cells (Tsukaya et al. 1993). Mutation in *CURLEY LEAF* (CLF) gene produced curled leaves in Arabidopsis (Kim et al. 1998). Increase in free vein ending, open venation pattern and rounded leaf structure were caused by *rotunda 1* (RON1) (Robles et al. 2010). Mutation in the *Scarecrow* gene in maize showed increase in number of BSCs, unusual differentiation of BS chloroplast, decrease in minor veins and alteration in vein density (Slewinski et al. 2012). These studies related to the abnormal vein patterning caused by mutation of particular genes provide some clue as to how Kranz anatomy is regulated and suggest involvement of multiple pathways in development of the Kranz pattern. The SCARECROW/SHORTROOT regulatory network has been determined to be one of the important components required for Kranz anatomy patterning because the leaves of C3 plants with mutated *Scarecrow* gene was normal, while in the C4 plants mutation in the same gene damaged the Kranz anatomy (Slewinski et al. 2012; Wang et al. 2013b). Recently, it has been shown that introduction of maize chromosomes into oat could increase the BSC size and reduce vein spacing in C3 oat leaves demonstrating that the anatomy of C3 leaf can be modified (Tolley et al. 2012). Moreover, a large effort has been put to screen sorghum (C4) mutants with increased vein spacing and rice (C3) mutants with reduced vein spacing so that the genes controlling vein spacing trait can be identified (Rizal et al. 2012).

### The activity of the Calvin cycle should be significantly reduced in MC and greatly enhanced in the BSC of rice

**Figure 3:**
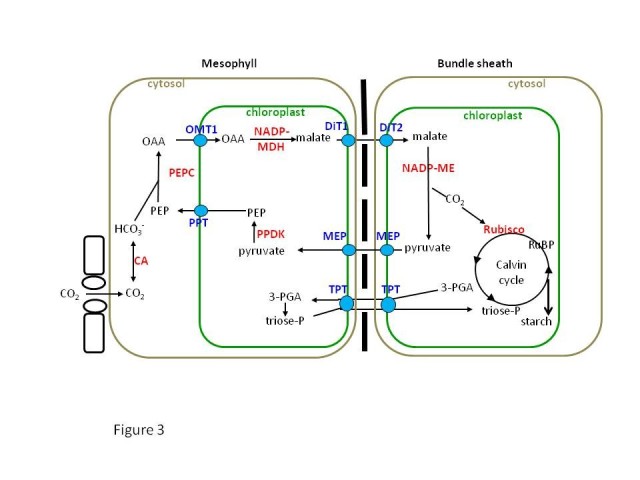
**Simplified biochemical pathway of NADP-ME subtype of C4 photosynthesis that is being genetically engineered into indica rice variety by the C4 rice consortium.** PEPC does the first carboxylation in the MC producing oxaloacetate which is further converted to malate by MDH. This C4 acid is transported from MC to the BSC chloroplasts where it is decarboxylated by NADP-ME to pyruvate and CO_2_ is released to Rubisco to carry out the Calvin cycle reactions. In C4 rice, Rubisco should be expressed in BSC and hence the increased CO_2_ levels at its site will reduce its oxygenation activity subsequently reducing the photorespiration. 3-PGA: 3-Phosphoglycarate, CA: Carbonic anhydrase, DiT1: Dicarboxylate translocator1, DiT2: Dicarboxylate translocator2, MEP: Mesophyll envelope protein, NADP-MDH: NADP-Malate dehydrogenase, NADP-ME: NADP-malic enzyme, PEP: Phospho*enol* pyruvate, OAA: Oxaloacetate, OMT: Oxoglutarate/malate translocator, PEPC: Phospho*enol* pyruvate carboxylase, PPDK: Pyruvate orthophosphate (Pi) dikinase, PPT: Phospho*enol* pyruvate phosphate translocator, Rubisco: Ribulose-1,5-bisphosphate carboxylase/oxygenase, RuBP: Ribulose-1,5-bisphosphate, and TPT: Triose-phosphate phosphate translocator.

C4 photosynthesis is characterized by a biochemical CO_2_ pumping mechanism that elevates the concentration of CO_2_ at the site of Rubisco. A high level of CO_2_ around Rubisco reduces the rate of photorespiration and increases net CO_2_ assimilation leading to highly efficient photosynthesis Weber and von (Caemmerer 2010). To achieve this, the CO_2_ assimilation in C4 is distributed over two cell types, the MCs and BSCs (Figure 3). Therefore, C4 carbon fixation depends on cell-specific gene expression and localization. The neighboring photosynthetically active BS and M cells interact to eliminate Rubisco-catalyzed O_2_ fixation. In two-celled type C4 plants, CO_2_ is at first fixed into the C4 acid named as oxaloacetate in MCs by an O_2_-insensitive carboxylase called phospho*enol* pyruvate carboxylase (PEPC, EC 4.1.1.31). The oxaloacetate is then converted to malate or aspartate and is transported to BSCs where it is decarboxylated and the CO_2_ is released. This CO_2_ is refixed by Rubisco and all the subsequent activities of Calvin cycle take place in chloroplast of BSCs (Nelson and Langdale 1989). Consequently, to make functioning C4 rice, Rubisco activity has to be greatly reduced in MCs and increased in BSCs which then confines the Calvin cycle to the BSCs of rice, like in a C4 system. On the other hand, certain genes encoding the C4 enzymes such as β carbonic anhydrase (CA) and PEPC have to be over expressed in cytosol of MCs of rice in order to facilitate the primary CO_2_ fixation so that CO_2_ can be concentrated and supplied to Rubisco in the BSCs. The C4 cycle also involves extensive transport of metabolites across the chloroplast envelope membrane and plasmalemma of MC and BSC (Figure 3). As such, in addition to the core C4 enzymes namely CA, PEPC, pyruvate orthophosphate (Pi) dikinase (PPDK, EC 2.7.9.1), NADP-dependent malate dehydrogenase (NADP-MDH, EC 1.1.1.82) and NADP-dependent malic enzyme (NADP-ME, EC 1.1.1.40), C4 pathway also requires insertion of metabolite transporters for oxaloacetate, malate, triose-phosphate and pyruvate into rice to provide increased transport capacity for the C4 cycle intermediates so that the Calvin cycle can function effectively in the BSCs (Weber and von Caemmerer 2010).

### The photorespiration in mesophyll cells has to be greatly reduced

In C3 plants carbon fixation and Calvin cycle take place in MCs. During carbon fixation, ribulose- 1, 5- bisphosphate (RuBP) - a five carbon compound, catalyzed by an enzyme ribulose-1,5-bisphosphate carboxylase oxygenase (Rubisco, EC.4.1.1.39) reacts with CO_2_ to form two molecules of 3-carbon compound called the 3-phosphoglycerate (3-PGA). Within the Calvin cycle, the two PGA molecules form an energy rich molecule of sugar (triose phosphate) and regenerates RuBP for the next cycle. At current atmospheric CO_2_ concentrations (ca. 400 ppm) Rubisco also catalyses a reaction between RuBP and O_2_ resulting in one molecule each of 2-phosphoglycolate and 3-PGA (Peterhansel and Maurino 2011). The 2-phosphoglycolate has to be converted back to 3-PGA through the process called photorespiration, which involves a series of biochemical reactions. During this process a loss of previously fixed carbon and nitrogen occurs and extra energy must also be used (Sharpe and Offermann 2013).

**Figure 4:**
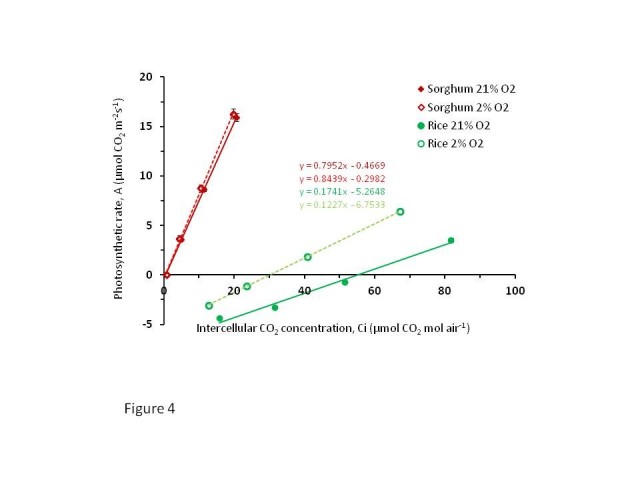
**Rate of photosynthesis in C3 and C4 at two different (21%** **and 2%** **) O**_**2**_**levels.** The rate of photosynthesis or the CO_2_ assimilation rate (A) was measured at intercellular CO_2_ concentration of 0, 20, 50, 100 and 200 μmol mol^-1^ altered at an interval of three minutes. The temperature of the block and the leaf was 28 ± 1°C, relative humidity was maintained at 68 ± 5%, constant light intensity of 1500 μmol m^-2^ s^-1^ and the flow rate was maintained at 400 μmol s^-1^.

C4 plants have developed mechanisms to restrict localization and activities of Rubisco in BSCs. MCs spatially prevent the contact between Rubisco in BSCs and O_2_ in the intercellular spaces, thus preventing loss of energy through photorespiration. The elimination of photorespiration by C4 plants is evidenced by their very low CO_2_ compensation point which is nearly zero and constantly high carboxylation efficiency (CE) without responding to the changes in O_2_ concentrations (Figure 4). In contrast, in C3 plants, with the change in O_2_ concentration from 21% to 2%, the compensation point significantly decreased from 55 to 30 ppm (Table 1). In Figure 4, the CE was calculated according to (Li et al. 2009) which showed that CE of sorghum did not significantly change with the change in O_2_ level, but in rice there was a highly significant improvement in CE when the level of O_2_ was decreased from 21 to 2% (Figure 4 and Table 1). The increase in CE in sorghum was just 6.1% while that in rice was 41.5% with the decrease in intercellular O_2_ concentration to 2% (Table 1). These data show that, there is a great potential to increase the photosynthetic capacity of rice by decreasing the photorespiration which in turn would increase the yield substantially. One way to reduce photorespiration in MC is by reducing the glycine decarboxylase (GDC) protein in MC and restrict its accumulation in BSC so that the decarboxylation of glycine occurs exclusively in BSC, thereby generating higher CO_2_ concentration in BSC, similar to that in C3-C4 intermediates (Monson and Rawsthorne 2000). The C4 rice consortium is testing this approach by using artificial microRNA designed against rice GDC-H subunit which is driven by *ZmPEPC* promoter (Kajala et al. 2011). Such a biochemical mechanism requires cellular specialization of the BSCs which include an increase in chloroplast numbers and mitochondria enriching the organelle content of the rice BSCs to help in recapture of the CO_2_ released by the decarboxylation of glycine by GDC (Ueno 2011). Another approach that was successful to capture the CO_2_ released by photorespiration to the site of photosynthesis is by transfer of *Escherichia coli* glycolate catabolic pathway to chloroplasts of *Arabidopsis thaliana* in which glycolate in chloroplast was directly converted to glycerate (Kebeish et al. 2007). This strategy which reduced photorespiration and enhanced photosynthesis in Arabidopsis involved stepwise nuclear transformation with five chloroplast targeted bacterial genes encoding glycolate dehydrogenase, glyoxylate carboligase and tartronic semialdehyde reductase could be applied to other C3 plants such as rice, however, the use of bacterial genes may not be preferred in engineering of C4 rice.

**Table 1:** **Differences in carboxylation efficiency (CE) and CO**_**2**_**compensation point (CP) between rice (C3) and sorghum (C4) at 21 and 2**% **oxygen level**

	CE (mol mol^-1^)		Increase in CE when O_2_level was reduced from 21 to 2%	CO_2_CP (mol mol^-1^)	
	21% O_2_	2% O_2_	(%)	21% O_2_	2% O_2_
Rice	0.123	0.174	41.5	54.877	30.241
Sorghum	0.795	0.844	6.1	0.587	0.365

### Engineering of C4 pathway into rice

It was thought that single cell C4 system could be faster to install in C3 plants. There are attempts to engineer single cell C4 photosynthesis system in rice too (Miyao et al. 2011). To introduce single cell C4–like pathway in which MC is made to capture and release CO_2_ in the manner it takes place in *Hydrilla verticillata* (L.f) Royle., four enzymes (PEPC, PPDK, NADP-MDH, and NADP-ME) involved in the pathway were overproduced in the transgenic rice leaves (Ku et al. 1999; Fukayama et al. 2001; Tsuchida et al. 2001; Taniguchi et al. 2008). A few of the major problems encountered that need to be addressed to make a single-cell C4-like pathway in rice are: mechanism to facilitate transport activity of PEP across the chloroplast envelope, the import of OAA into the chloroplasts and the direction of the NADP-ME reaction, involvement of NADP-MDH, the presence of endogenous PEPC inside the MC chloroplast of rice and further elevation of NADP-MDH activity were reported to be necessary (Miyao et al. 2011). Terrestrial single cell C4 species such as *Bienertia cycloptera, B. sinuspersici* and *Suaeda aralocaspica*, belonging to Chenopodiaceae family also need spatial compartmentalization of the carbon assimilation and decarboxylation (Chuong et al. 2006). These species have dimorphic chloroplasts in those compartments. The earlier attempts produced futile cycle which was due to no change in anatomy, lack of appropriate transporters and the maize genes transformed into rice were not appropriately expressed in cell specific manner and were not regulated like in maize but were regulated like the endogenous rice C3 isoforms (Miyao et al. 2011).

To engineer the photosynthetic pathway from C3 to C4 within two decades, which took million of years in nature, C4 rice consortium began the simultaneous gene discovery and engineering of already known genes into rice aiming to form C4 rice with Kranz type anatomy. C4 genes such as *CA*, *PEPC*, *PPDK*, *NADP-ME*, and *NADP-MDH* are cloned from maize and transformed into rice. Also the transporters that were over expressed in the C4 metabolic pathways such as 2-oxoglutarate/malate transporter (OMT1), dicarboxylate transporter1 (DiT1), dicarboxylate transporter2 (DiT2), PEP/phosphate transporter (PPT1), mesophyll envelope protein (MEP) and triose-phosphate phosphate translocator (TPT) that were recently identified through proteomics of maize BS and MS cells (Friso et al. 2010) are being transformed into rice (Figure 3). The C4 rice consortium members are also involved in discovering novel genes related to Kranz anatomy (Wang et al. 2013b). Once tested, the promising candidate genes controlling the Kranz anatomy will also be introduced in the rice plants that have been engineered with the C4 biochemical pathway genes.

## Conclusion

The C4 photosynthetic pathway has evolved more than 66 times in different species, suggesting that C3 plants may be in some way preconditioned to C4 photosynthesis. Developing crop plants with enhanced photosynthesis will improve crop yield and make efficient use of resources in a sustainable manner. The C4 rice consortium is striving to install a maize-like photosynthetic mechanism in rice to break its yield barrier and to breed a new generation of “climate-ready” rice which will yield more even under the situations of increasing temperature and decreasing water availability. This initiative demands a holistic approach of understanding the regulatory networks and the complex mechanism underlying the C4 photosynthetic pathway and their systematic introduction into the rice genome.

## Authors’ original submitted files for images

Below are the links to the authors’ original submitted files for images. Authors’ original file for figure 1 Authors’ original file for figure 2 Authors’ original file for figure 3 Authors’ original file for figure 4
