# Supplementary figures and images for: Improvement of photosynthesis in rice (Oryza sativa L.) by inserting the C4 pathway
Source: Rice (N Y). 2013 Oct 28;6:28. doi: 10.1186/1939-8433-6-28 (PMC4883725; doi:10.1186/1939-8433-6-28)

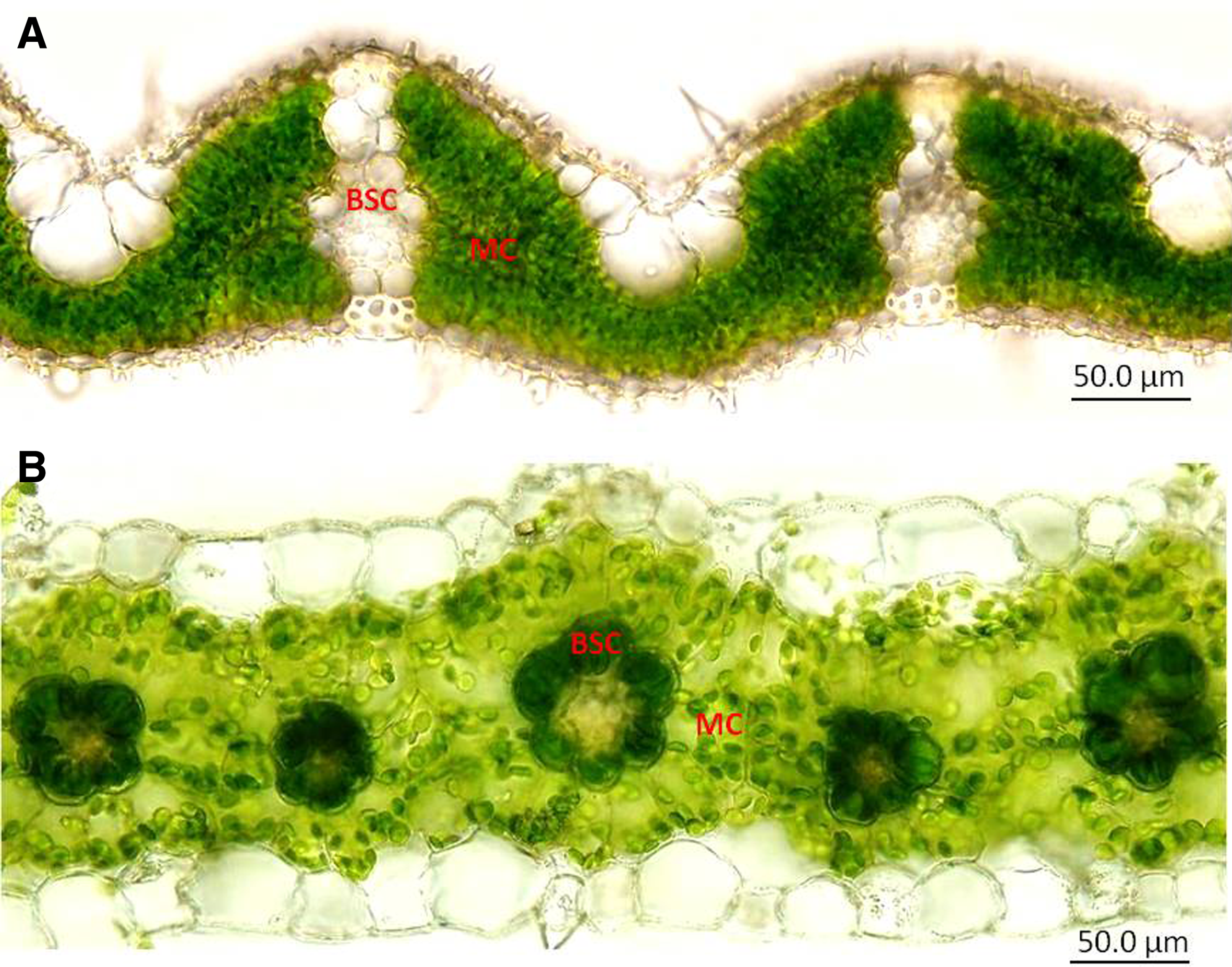

Supplement: Supplementary file 1 — Authors’ original file for figure 1 [file 12284_2013_78_MOESM1_ESM.tif]

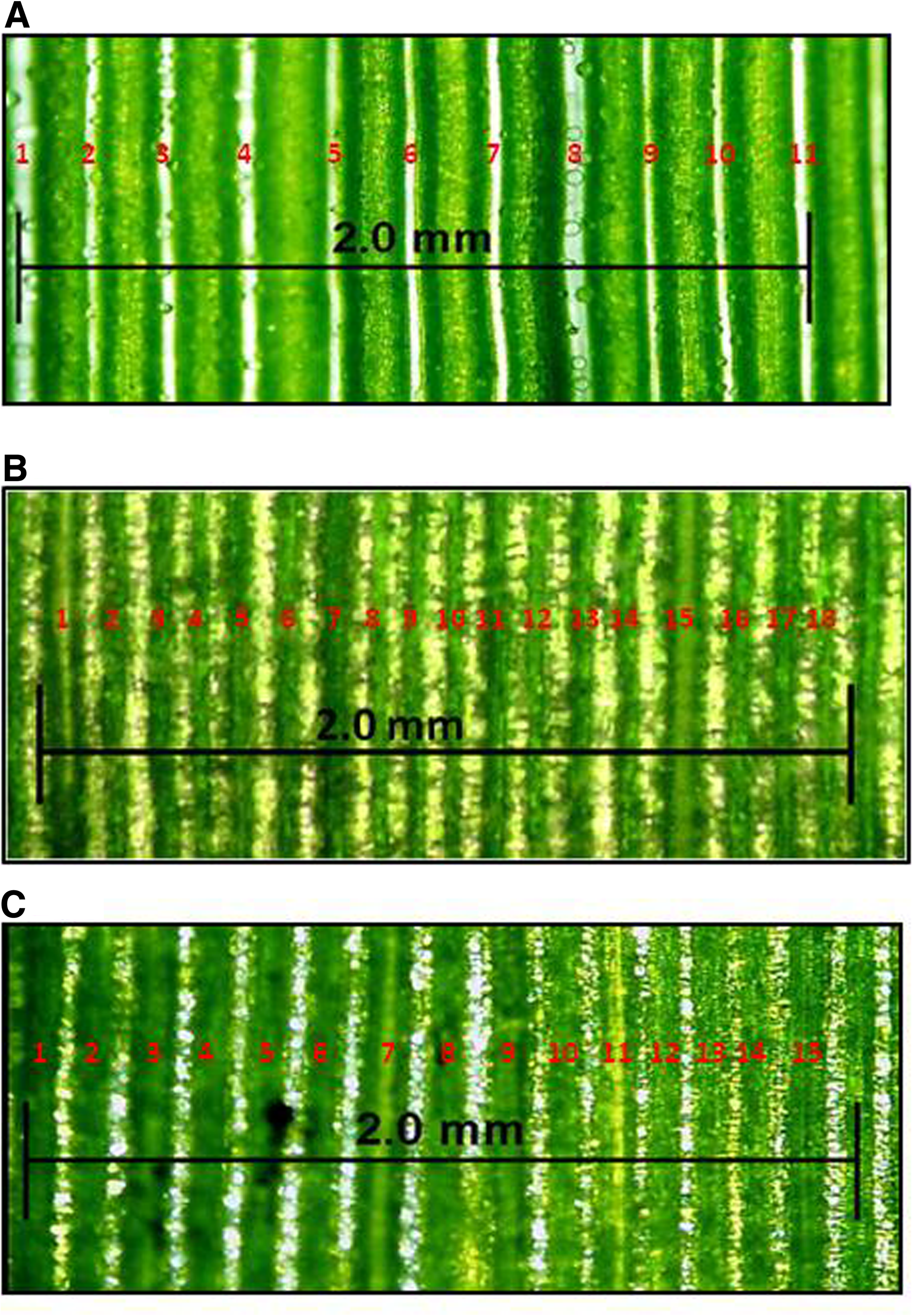

Supplement: Supplementary file 2 — Authors’ original file for figure 2 [file 12284_2013_78_MOESM2_ESM.tif]

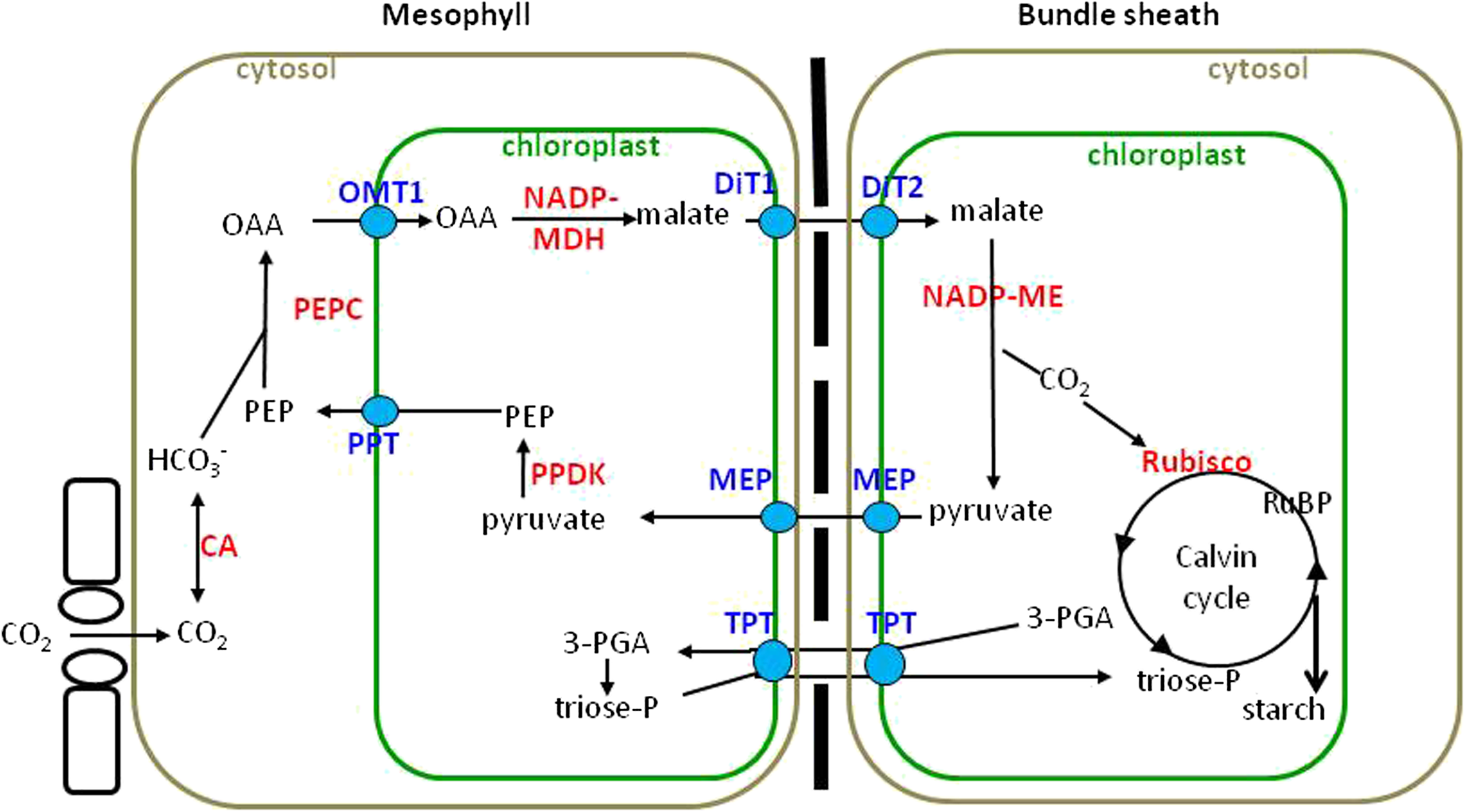

Supplement: Supplementary file 3 — Authors’ original file for figure 3 [file 12284_2013_78_MOESM3_ESM.tif]

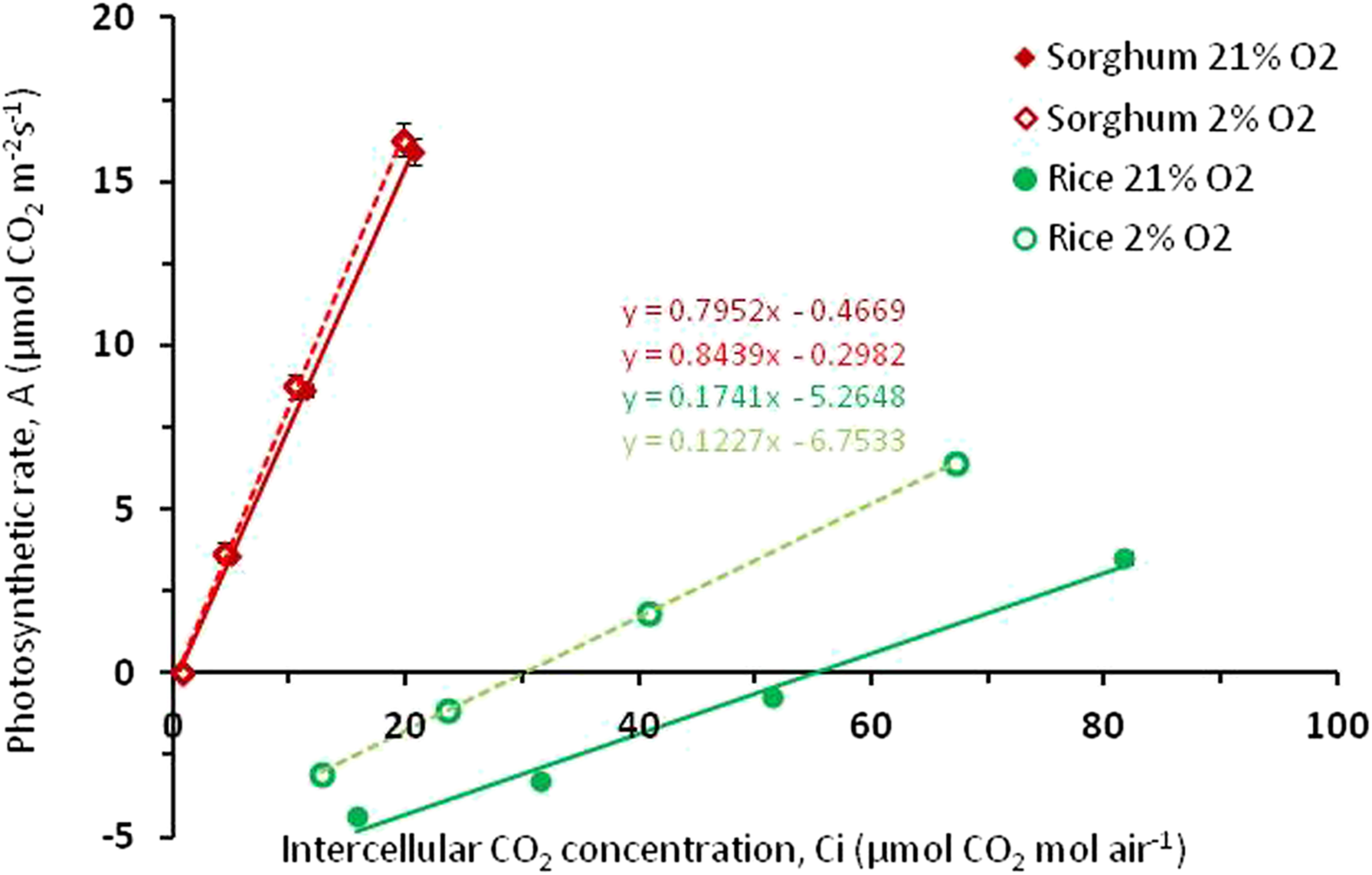

Supplement: Supplementary file 4 — Authors’ original file for figure 4 [file 12284_2013_78_MOESM4_ESM.tif]
